# Charged Biological Membranes Repel Large Neutral Molecules by Surface Dielectrophoresis and Counterion Pressure

**DOI:** 10.1021/jacs.3c12348

**Published:** 2024-01-16

**Authors:** Marcel Aguilella-Arzo, David P. Hoogerheide, Mathieu Doucet, Hanyu Wang, Vicente M. Aguilella

**Affiliations:** † Laboratory of Molecular Biophysics, Department of Physics, Universitat Jaume I, 12071, Castellón, Spain; ‡ Center for Neutron Research, National Institute of Standards and Technology, Gaithersburg, Maryland 20899, United States; § Neutron Scattering Division, Oak Ridge National Laboratory, Oak Ridge, Tennessee 37831, United States; ∥ Center for Nanophase Materials Sciences, Oak Ridge National Laboratory, Oak Ridge, Tennessee 37831, United States

## Abstract

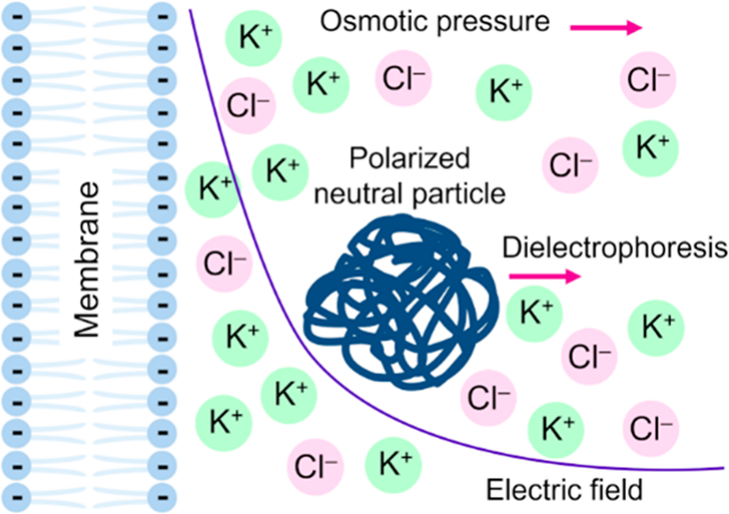

Macromolecular crowding is the usual condition of cells. The implications of the crowded cellular environment for protein stability and folding, protein–protein interactions, and intracellular transport drive a growing interest in quantifying the effects of crowding. While the properties of crowded solutions have been extensively studied, less attention has been paid to the interaction of crowders with the cellular boundaries, i.e., membranes. However, membranes are key components of cells and most subcellular organelles, playing a central role in regulating protein channel and receptor functions by recruiting and binding charged and neutral solutes. While membrane interactions with charged solutes are dominated by electrostatic forces, here we show that significant charge-induced forces also exist between membranes and neutral solutes. Using neutron reflectometry measurements and molecular dynamics simulations of poly(ethylene glycol) (PEG) polymers of different molecular weights near charged and neutral membranes, we demonstrate the roles of surface dielectrophoresis and counterion pressure in repelling PEG from charged membrane surfaces. The resulting depletion zone is expected to have consequences for drug design and delivery, the activity of proteins near membrane surfaces, and the transport of small molecules along the membrane surface.

## Introduction

Mimicking the cell environment involves reproducing experimentally the crowding conditions typical of the cell and its organelles.^1,2^ While the actual macromolecule concentration depends on the type of cell or subcellular organelle, crowding affects not only interactions between molecules in solution but also interactions between those molecules and the ubiquitous biological membranes that form cellular boundaries. Given the number of transmembrane proteins that act as signal receptors and the fact that membrane recruitment is frequently a rate-limiting step to protein channel regulation by cytosolic proteins,^3^ the forces governing these interactions are of high physiological relevance. Experimentally, synthetic polymers are frequently used to mimic crowders. Poly(ethylene glycol) (PEG) is an electrically neutral polymer commonly substituted for naturally occurring biopolymers and proteins because of its high solubility in both water and nonpolar solvents. PEG is also widely used in many different practical contexts, including the pharmaceutical and food industries,^4^ protein precipitation,^5,6^ and biomedical applications involving drug delivery through PEGylation.^7^ PEG is also a versatile biophysical toolbox to induce osmotic stress in polymer-excluded regions^8,9^ or to probe protein ion channels to get information about their size^10^ or their access resistance.^11,12^

Many of these applications involve the proximity of neutral polymers to charged interfaces or their confinement in charged pores with nanometer dimensions. In such nanoscopic systems, charged surfaces unexpectedly appear to repel neutral polymers; for example, the charge of a protein channel may change the partitioning equilibrium of PEG molecules between the adjacent ionic solution and the pore.^13^ This effect is thought to be due to repulsive dielectrophoretic and osmotic forces on the polymer^14^ and has been observed under a variety of dilution conditions.^15^ Direct evidence of this hypothesis has so far remained elusive. Dielectrophoresis (DEP)^16^ is typically observed in the ballistic motion of micron-scale or larger particles in electric field gradients when the particle’s dielectric properties differ from those of the surrounding medium; however, these gradients are typically not of sufficient magnitude to affect nanoscale particles.^17,18^

At nanometer distances from a charged surface, however, electric field gradients are extremely large, nearly 4 orders of magnitude larger than those employed in DEP applications. Here we show that these extreme electric field gradients give rise to a new manifestation of DEP: surface dielectrophoresis (sDEP). Along with counterion pressure effects,^19^ sDEP yields a sufficiently large repulsive net force to exclude neutral PEG molecules—and by extension neutral biological macromolecules—from the solution closest to the surface.

We demonstrate the effects of sDEP from three perspectives. First, we derive analytical expressions for the PEG free energy arising from both contributions by assuming a simplified spherical model of the PEG molecule with low permittivity and explore the influence of the PEG molecular weight (MW) on the depletion effect. Second, we perform NR measurements from surfaces decorated with single bilayer lipid membranes in physiological ionic strength solutions containing PEG concentrations within the dilute regime. The PEG volume fraction that follows from the neutron scattering length density (nSLD) spatial profile provides experimental evidence of an exclusion region near both negatively and positively charged lipid bilayers that is substantially larger than that observed for neutral bilayers. Third, we perform all-atom molecular dynamics (MD) simulations to calculate the potential of mean force (PMF) of single PEG molecules of varying MW near-neutral 1,2-dipalmitoyl-phosphatidylcholine (DPPC), negatively charged 1,2-dipalmitoyl-phosphatidylserine (DPPS), and positively charged 1,2-dipalmitoyl-3-trimethylammonium-propane (DPTAP) planar lipid bilayers in solutions of physiological concentration; we also show that MD simulations of a system with many PEG molecules (10% w/w PEG600 in a 0.1 M KCl buffer) also display a depletion of the PEG mass fraction near the charged membrane. The decay of the PEG self-energy with the distance from the charged lipid headgroups is consistent with the analytical predictions and supports the depletion layer evidence gained from NR measurements.

## Theory

**Figure 1:**
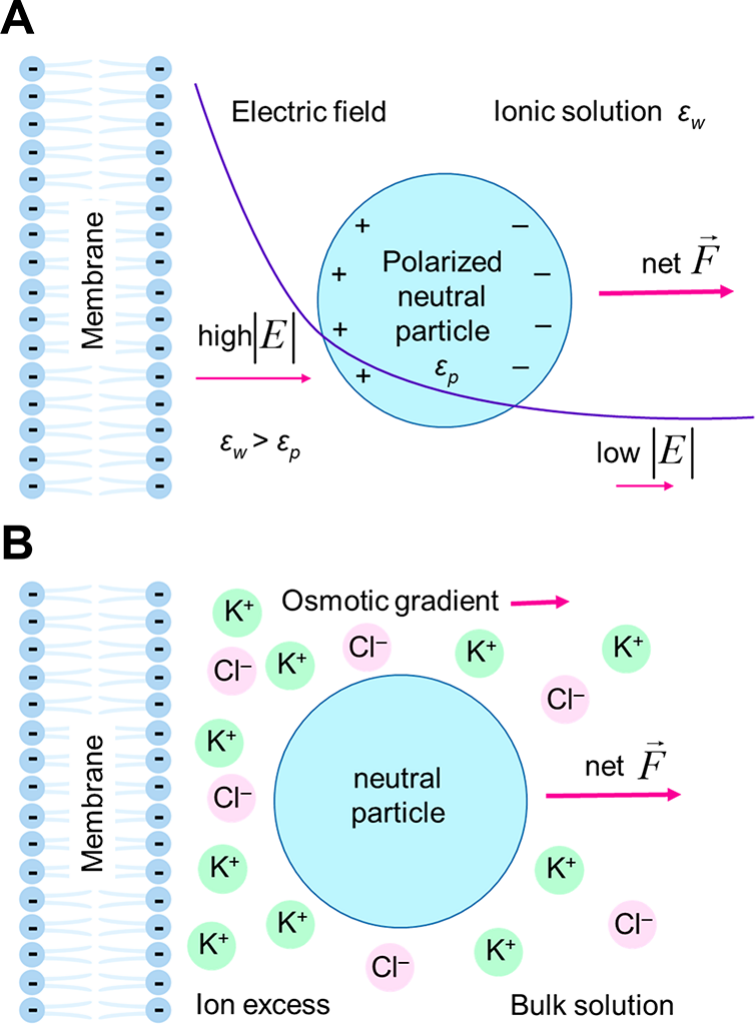
(A) Surface dielectrophoretic (sDEP) interaction. Polarization forces acting on a neutral particle in a nonuniform electric field are created by a charged membrane bathed by an ionic solution. The net force is repulsive when the dielectric constant is lower in the particle than in the surrounding medium. (B) Excess ion density near a charged membrane induces an osmotic pressure on a neutral particle that approaches the electric double layer.

First, let us consider the effect of the dielectrophoretic force **F**_D_ acting on neutral polymers in close proximity to charged biological interfaces. PEG is a neutral molecule with much lower polarizability^20^ than water, and the electric double layer of a charged interface in an ionic solution gives rise to a nonuniform electric field close to the interface. Because PEG has a lower dielectric constant than water, the net dielectrophoretic force is directed away from the charged surface (Figure 1A). In addition, as the PEG excluded volume overlaps with the electric double layer, the hydrostatic pressure acts as another repelling force, **F**_H_, on the PEG molecule. This force can be derived from the Navier–Stokes equation, considering an electrical body force, as shown in Figure 1B.

### Dielectrophoretic Force

The classical expression for the DEP force acting on a neutral, homogeneous, spherical particle of radius *a* in a static electric field is^21^
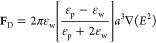
1where ε_p_ and ε_w_ are the absolute permittivities of the particle and the solvent, respectively. *E* is the modulus of the electric field. The fraction enclosed in brackets is the Clausius–Mossotti factor, which is related to the effective polarizability of the particle. This factor is negative when the particle is immersed in a medium of higher polarizability (ε_p_ < ε_w_), which implies a force pushing the particle toward lower field regions (Figure 1A). Despite its widespread use in many applications, eq 1 is based on several approximations. The key ones are (a) representing the polarized particle by a single dipole whose moment is obtained by assuming the field is uniform and (b) assuming the solvent contains no mobile charges. Here we derive an expression for the DEP force **F**_D_ in ionic solutions for neutral dielectric particles whose size is comparable to the characteristic Debye length of the solution (∼1 nm for physiological ionic strength). To get **F**_D_ we integrate the Maxwell stress tensor over the particle boundary surface *S*_p_. Following Cai et al.^22^ the integration can be simplified to
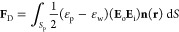
2where **E**_o_ and **E**_i_ are the electric field outside and inside the particle boundary surface and **n**(**r**) is the unit normal vector pointing away from that boundary. To proceed with the integration, as a first approximation, we consider the PEG molecule as a neutral sphere of radius *a*. The general solution to the Laplace equation within its volume under azimuthal symmetry can be expressed in terms of Legendre polynomials. The charge distribution outside the sphere is described by the Poisson–Boltzmann equation (using spherical coordinates) for a solution of Debye length κ^–1^ in contact with a surface (the membrane) with a surface charge density σ. The boundary conditions for the electric potential and the electric field over the particle surface involve rather cumbersome expressions (see Supporting Information), but we can reach a closed expression for the DEP force modulus as a series expansion. We are interested in the *z*-component of the DEP force, i.e., normal to the charged plane. The dielectrophoretic free energy of the particle as a function of the distance *z* to the charged interface can be expressed as the work needed to bring the particle from infinity to *z*. Then, by grouping particle radius, ionic strength, surface charge density, and permittivities into the prefactor *K*_D_ (see Supporting Information for details), the dielectrophoretic free energy *E*_D_ of the particle as a function of the distance *z* to the charged interface becomes
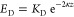
3

The classical expression for *E*_D_ that follows from eq 1 underestimates the dielectrophoretic energy compared to that obtained from eq 3. This new expression for *E*_D_ is valid for ionic solutions.

### Hydrostatic Force

The ion excess pressure on the particle where it overlaps with the electric double layer gives rise to a repulsive hydrostatic force that can be obtained by integrating the pressure tensor^19^ over the particle boundary surface:
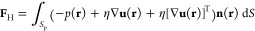
4where η is the viscosity of the fluid, **u**(**r**) is the solvent velocity field, and *p*(**r**) is the local pressure, related to the local charge density through the Poisson–Boltzmann equation. Linearizing the electric potential allows integration of *p*(**r**) over the particle boundary (see Supporting Information), yielding a closed expression for the *z* component of the hydrodynamic force **F**_H_. Analogously to the dielectrophoretic force, **F**_H_ can be expressed as a prefactor by the functional dependence on distance *z*. Then, the corresponding free energy *E*_H_ of the particle separated a distance *z* from the charged membrane becomes
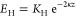
5The overall repulsive force **F**_H_ + **F**_D_ acting on the particle increases with the particle’s size and becomes stronger as the particle approaches the charged surface. To estimate an average distance of the particle closest approach, i.e., the size of the region where it is likely excluded, we assume that the depletion layer spans a region where the particle free energy (adding up both contributions) exceeds the thermal energy, *k*_B_*T*. By assuming as a first crude assumption that the PEG molecule can be represented by a sphere with PEG hydrodynamic radius, *R*_h_(23) and low permittivity ε_p_ = 10ε_o_,^20^ we can estimate the average closest approach of a PEG molecule to a negatively charged DPPS membrane in a solution of physiological concentration (0.1 M KCl). This distance *d* is several times larger than the PEG particle radius for low-to-medium MW PEGs as shown in Table S1. Both the distance *d* to the PEG mass center and the width of the effective “PEG-free” solution layer, d* = *d* – *R*_h_, increase with the PEG MW. Although some of the assumptions made to reach analytical expressions (a polymer chain as a neutral compact sphere of low permittivity) are unrealistic, these expressions provide a first rough estimation of the combined effect of sDEP and ion excess pressure and can be used to estimate qualitatively the influence of the particle size, the ionic strength of the medium, and the membrane surface charge density on the particle–membrane interaction. For the low-to-medium MW PEGs near a DPPS membrane considered, the hydrostatic contribution exceeds the dielectrophoretic contribution (Figure S1).

## Results

### Neutron Reflectometry (NR)

To observe dielectrophoretic exclusion experimentally, we turned to NR, which is an isotope-sensitive interfacial technique that has good contrast between hydrogen- and deuterium-containing materials—in this case, between hydrogenated PEG and heavy (deuterated) water. For these experiments, solid supported bilayer lipid membranes (ssBLMs) on a naturally SiO_2_-terminated Si substrate film were chosen due to their ease of fabrication and high, lipid-dependent charge density.

**Figure 2:**
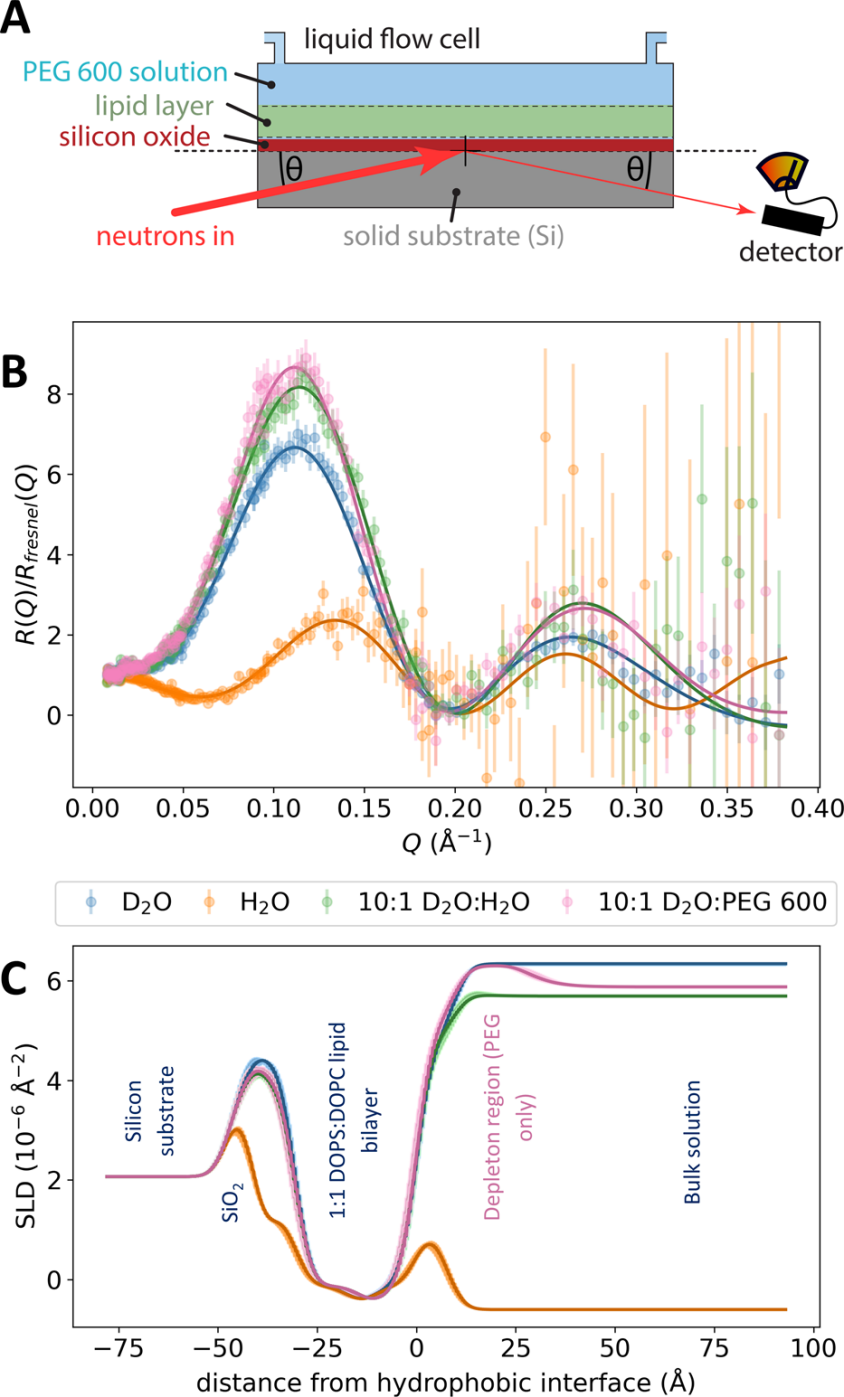
Neutron reflectivity data for a 1:1 DOPC:DOPS lipid bilayer supported on a silicon substrate and exposed to 10:1 D_2_O:PEG600 by weight (100 mM KCl, 10 mM tris, pH 7.4). (A) Schematic of the neutron reflectometry experiment. (B) Neutron reflectivity scaled to the Fresnel reflectivity, i.e., the reflectivity expected between that solution and silicon in the absence of any interfacial structure. Each curve represents the reflectivity of the surface when the reservoir is filled with the solution composition indicated. Error bars are 68% confidence intervals estimated from Poisson counting statistics. (C) Neutron scattering length density (nSLD) profiles of the interface. Shading shows 68% and 95% confidence intervals.

NR experiments were carried out using a liquid flow cell shown schematically in Figure 2A and described in the Experimental Section. The reflectivity of neutrons from the ssBLM-decorated interface was first measured in the absence of PEG in pure D_2_O and H_2_O buffer (100 mM KCl, 10 mM tris, pH 7.4); then, the surface was exposed to a 10:1 D_2_O buffer:PEG600 solution. NR patterns for each condition are shown in Figure 2B. Each pattern is normalized to the Fresnel reflectivity —the reflectivity from a hypothetical single interface between silicon and the bulk solution— to emphasize the differences among the reflectivity patterns.

The NR data were then fit to a composition space model, as described in the Experimental Section. The composition space model positions the various molecular groups (bilayer headgroups, bilayer acyl chains, and PEG) in a thin film structure, accounting for molecular volumes, connectivity, and stoichiometry. Any remaining space is filled with solvent. The model parameters adjust the physical locations of the various molecular components. From the known molecular volumes and elemental compositions of each component in the structure, a nSLD profile describing the model thin-film structure can be calculated. The nSLD profile directly leads to a predicted NR pattern via a Fourier transformation.

The predicted NR patterns corresponding to the optimized models are shown as solid curves in Figure 2B, and the corresponding nSLD profiles are shown in Figure 2C. The interface between the lipid tails and headgroup regions for the outer leaflet is at *z* = 0. At high *z*, above the interface, the nSLD directly yields the fraction of PEG (nSLD = 0.7 × 10^–6^ Å^–2^) in the D_2_O solution (nSLD ≈ 6.4 × 10^–6^ Å^–2^). Approaching the surface in the negative z direction, the SLD trends slowly toward the nSLD of D_2_O, confirming the presence of a region where PEG is excluded from near the surface, before sharply decreasing in the lipid bilayer (nSLD ≈ −0.3 × 10^–6^ Å^–2^). A control measurement on the same surface using a 10:1 D_2_O:H_2_O solution, which has a similar bulk nSLD, shows no such depletion region.

**Figure 3:**
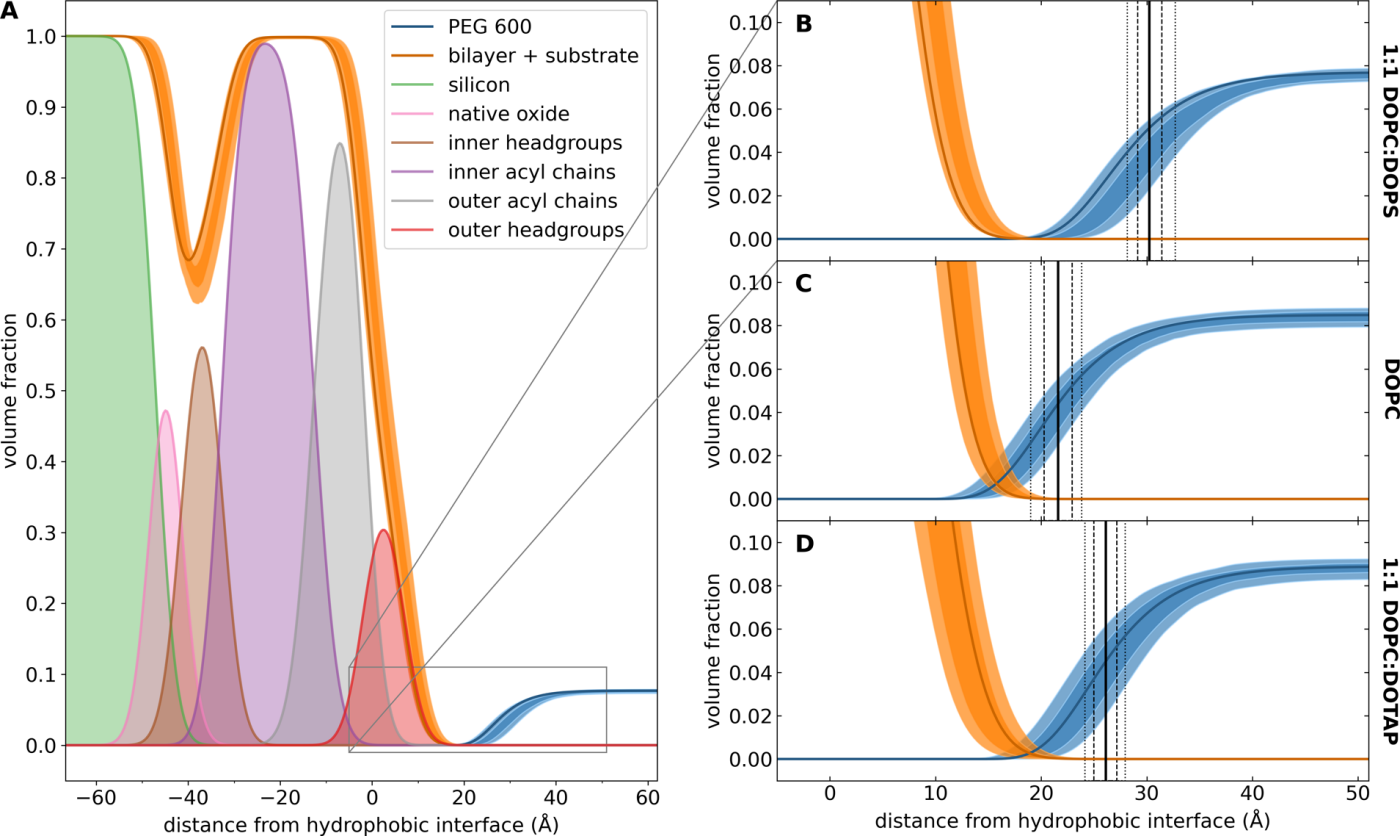
Composition space analysis of NR data for 10:1 D_2_O:PEG600 by weight (100 mM KCl, 10 mM tris, pH 7.4). (A) Volume occupancy profiles of each molecular component. For the bilayer + substrate sum and the PEG600 profile, shading shows the 68% and 95% confidence intervals, while the dark curve shows the best fit. (B–D) Detail of depletion region near the bilayer surface for three lipid compositions: 1:1 DOPC:DOPS (negatively charged), DOPC (neutral), and 1:1 DOPC:DOTAP (positively charged). Shading represents the 68% and 95% confidence intervals. The vertical solid line represents the median exclusion region, i.e., the distance from the hydrophobic interface at which the PEG600 density drops to half its solution density. The dashed and dotted lines represent the 68% and 95% confidence intervals of this value, respectively.

Figure 3 shows the volume occupancy decomposition of the scattering length density profiles. In Figure 3A, the volume occupancy distributions of each molecular component in the optimized composition space models are shown. The shaded curves show the uncertainties at the 68% and 95% confidence intervals for the entire substrate/bilayer complex as well as for the PEG600. Figure 3B shows a detail of the indicated region. The vertical solid line shows the extent of the exclusion region, i.e., the distance from the hydrophobic interface at which the PEG600 density drops to half its bulk value, while the broken lines show the 68% and 95% confidence intervals of the exclusion region, respectively. Figures 3C and 3D show the same results for separate data sets acquired using neutral DOPC and positively charged DOPC:DOTAP bilayers.

Table 1 shows the exclusion distance for each lipid composition and experimental condition, demonstrating a significant 6 Å increase in the depletion region for both charged bilayers relative to the neutral DOPC at 110 mM ionic strength and a nominal 10% by weight PEG600 preparation. The shift is independent of the sign of the charge, as expected from eqs 1 and 2.

**Table 1:** Extent of the Exclusion Region in 10:1 D_2_O:PEG600 Experiments with 68% Confidence Intervals

Ionic strength (mM)	Wt% PEG	Lipid composition	Exclusion region (Å)
110	4.8	1:1 DOPC:DOPS	34.8_–4.5_^+7.5^
110	4.8	DOPC	24.9_–1.5_^+1.5^
110	4.8	1:1 DOPC:DOTAP	29.7_–2.1_^+2.0^

110	9.1	1:1 DOPC:DOPS	29.9_–1.3_^+1.2^
110	9.1	DOPC	22.9_–1.1_^+1.3^
110	9.1	1:1 DOPC:DOTAP	28.1_-1.4_^+1.3^

110	16.7	1:1 DOPC:DOPS	19.70_–0.89_^+0.95^
110	16.7	DOPC	19.00_–0.82_^+0.81^
110	16.7	1:1 DOPC:DOTAP	21.31_–0.88_^+0.89^

10	9.1	1:1 DOPC:DOPS	21.4_-1.5_^+1.2^
10	9.1	DOPC	21.3_–1.4_^+1.3^
10	9.1	1:1 DOPC:DOTAP	25.8_–1.2_^+1.1^

Subsequent experiments on each bilayer, also tabulated in Table 1, show that the exclusion effect can be altered by changing the experimental conditions. At higher dilution (20:1 D_2_O:PEG600), the exclusion effect persists but is more difficult to measure due to the decreased contrast between the PEG solution and pure D_2_O (Figure S3 and Figure S4). At higher concentration (5:1 D_2_O:PEG600), the exclusion effect, if present, is smaller than the sensitivity of the measurement (Figure S5 and Figure S6). Under these conditions, moreover, the bilayer moves closer to the substrate, presumably due to dehydration of the bilayer by the concentrated PEG solution. Finally, 10:1 D_2_O:PEG600 solution at a low salt concentration (5 mM KCl, 5 mM tris, pH 7.4) also shows a diminished exclusion effect (Figure S7 and Figure S8).

To test whether the exclusion effect can be generalized to non-bilayer surfaces, we also acquired NR patterns from solutions of PEG6000 molecules in contact with a highly negatively charged SiO_2_ surface in a 10 mM ionic strength buffer (Figure S9A). Unlike in the bilayer case, in these experiments we were not able to tune the surface charge. Nonetheless, a significant exclusion region was observed (Figure S9B,C). Similarly to the bilayer experiments, the interaction strength with the surface *E*_0_ decreased with increasing density of the PEG6000 molecules (Figure S9C,D). An identical effect was observed on the LIQREF and CANDOR reflectometers.

### Molecular Dynamics (MD) Simulations

#### Free Energy of a Single PEG Molecule

**Figure 4:**
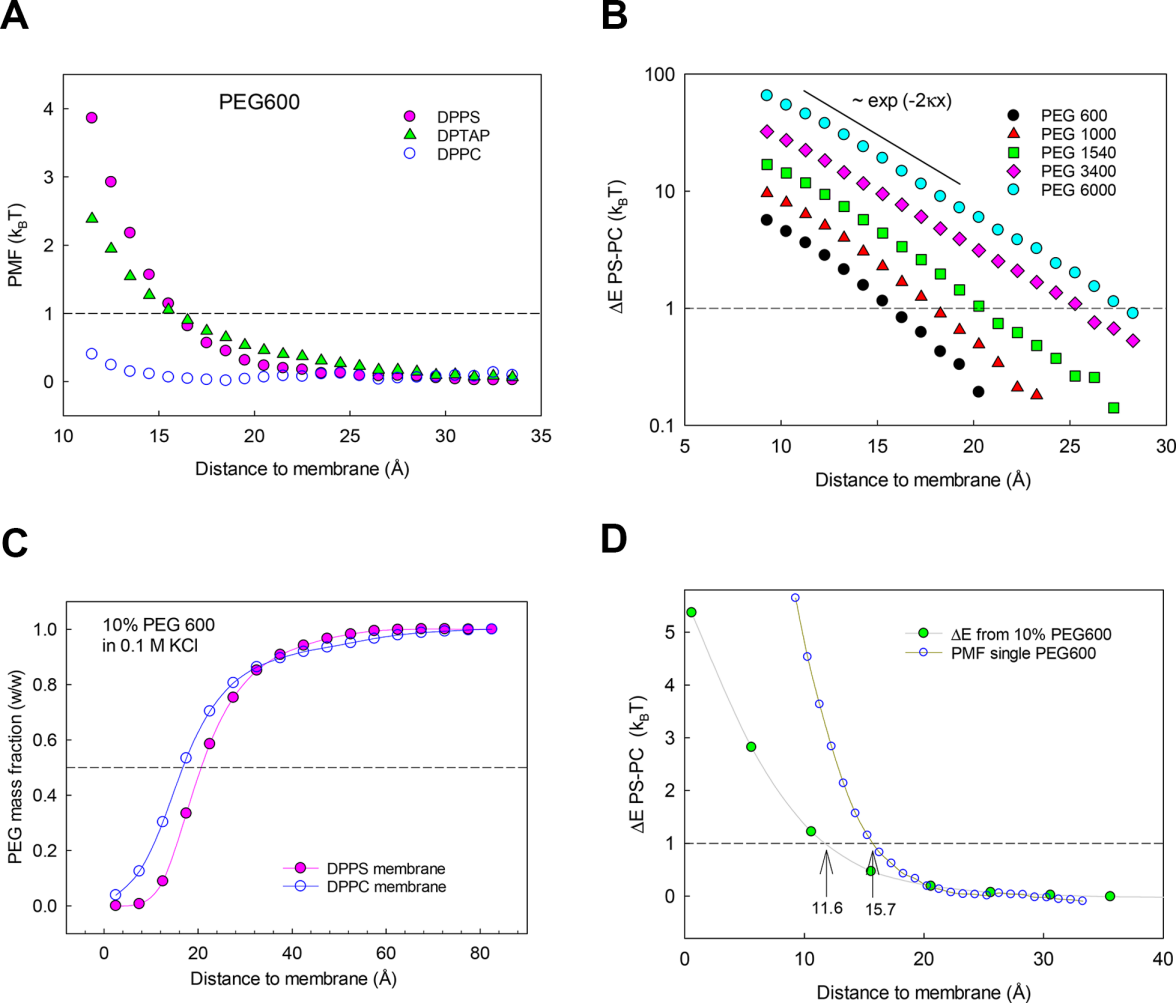
(A) Free energy (PMF) of a single PEG600 molecule in 0.1 M KCl approaching a negatively charged (DPPS), a positively charged (DPTAP) and a neutral (DPPC) membrane as labeled. The PEG exclusion region (where PMF > 1 *k*_B_*T*) is the same for both charged membranes, and it spans around 16 Å off the lipid charged headgroups. (B) Difference between PMF in DPPS and DPPC to keep only the relevant interaction (hydrostatic and dielectrophoretic) for PEGs of increasing size; it exhibits an exponential decay in accordance with theoretical prediction (eqs 3 and 5). The intersection with the dashed line shows an increasing size of the PEG exclusion region with the PEG MW. The plot for PEG 2000 has been omitted for clarity. (C) Normalized PEG600 mass fraction vs distance for 10% (w/w) PEG solutions in 0.1 M KCl for DPPS and DPPC membranes. (D) Comparison of PEG600 free energy difference between DPPS and DPPC obtained for a single PEG600 molecule and a 10% PEG solution. In single PEG simulations (panels A–D) the distance in the horizontal axis corresponds to the distance from the PEG mass center to the average *z*-position of the lipid charge. In 10% PEG simulations (panels C and D), the PEG mass center is replaced by the center of the bin used to average the PEG mass fraction.

We ran 1.6 μs long all-atom MD simulations of PEG single molecules of different MW (600, 1000, 1540, 2000, 3400 and 6000, corresponding to 13, 22, 35, 45, 77, and 136 monomers, respectively) in the presence of water molecules, K^+^ and Cl^–^ ions (at physiological concentration, 0.1 M), and planar lipid bilayers. The free energy of interaction (PMF) between the polymer and the membrane was extracted using the accelerated weight histogram (AWH) method.^24^ The PMF landscape was sampled as a function of the distance from the PEG mass center to the membrane-solution interface. The PMF values were very sensitive to the membrane net charge (Figure 4A). They exceeded the thermal energy when the PEG molecule approached a charged membrane, whereas they remained below 1 *k*_B_*T* in simulations with a neutral membrane. Interestingly, PEG free energies near a negatively charged membrane (DPPS) and a positively charged membrane (DPTAP) were similar, as expected from the theoretical prediction that the DEP effect does not depend on the sign of the electric field. The slight difference in free energy close to the lipid headgroups between oppositely charged membranes is likely due to the lower surface charge density of DPTAP (1/69.8 e/Å^2^ = 0.23 C/m^2^) compared to DPPS (1/46 e/Å^2^ = 0.35 C/m^2^), which according to the analytical expressions should decrease *E*_D_ for DPTAP relative to that for DPPS. Figure 4A shows the results of simulations run with PEG600, which are qualitatively the same for other larger PEGs. Interestingly, the distance *d* at which the interaction free energy is 1 *k*_B_*T* (∼16 Å, dashed line in Figure 4A) is roughly the same for DPPS and DPTAP membranes and is significantly larger than the polymer hydrodynamic radius (∼7 Å). Here, the distance from the membrane is the distance from the center of mass of the PEG molecule to the average *z*-position of the lipid charge (as defined in the Experimental Section).

To exclude all PEG free energy contributions which are already present near a neutral zwitterionic lipid membrane (entropic penalty, short-range interactions, restricted water orientation, etc.), we ran parallel simulations in charged and neutral membranes (with the same acyl chains) and subtracted the PEG free energy landscape of PEG in DPPC from that in DPPS or DPTAP membranes. We hypothesize that this energy shift Δ*E* should correspond to the sum of the dielectrophoretic *E*_*D*_ and hydrostatic *E*_H_ free energies. Figure 4B shows the field gradient contribution to Δ*E* obtained from simulations of PEGs with varying MW (600–6000). The PEG free energy difference Δ*E* at a given distance *z* from the membrane increases with MW and so does the extent of the region where the free energy is higher than the random thermal energy (shown by the intersection with the dashed line). The semilog scale in Figure 4B helps visualize the exponential decay length of the free energy, which the analytical expressions predict should be equal to half the Debye length (eqs 3 and 5). MD simulations yield the same qualitative picture as analytical predictions: a repulsive interaction of the neutral polymer from the charged interface that increases with the surface charge density and the polymer size. Interestingly, for a given distance to the membrane, this difference Δ*E* obtained from MD simulations scales with the third power of the PEG hydrodynamic radius, in agreement with analytical expressions (see Figure S2).

For PEG600, the free energy difference Δ*E* becomes 1 *k*_*B*_*T* at *d* ≈ 16 Å from the average z-position of the lipid charge, while the analytical prediction is *d* ≈ 21 Å (Table S1). This deviation can be attributed to the obvious simplification made before when integrating the Maxwell tensor and the pressure tensor over a solid and uniform, low permittivity sphere of radius *R*_h_. The extent of the polymer depletion region *d* increases with MW (Table 2). Note that all of the analytical predictions (Table S1) overestimate the corresponding values that arise from MD simulations.

**Table 2:** The Extent of the Polymer Depletion Region *d* Increases with MW

MW	*R*_h_ (Å)^a^	*d* (Å)^b^
600	7	16
1000	9	18
1540	11	20
2000	13	22
3400	17	26
6000	22	28

a Values from ref (23).

b PMF-AWH calculations of distance where free energy (DPPS-DPPC) becomes 1 *k*_B_*T*.

#### PEG Solutions

Typically, experiments of PEG equilibrium partitioning in protein channels^10^ or with PEG as crowding agent or osmotic stress inducer are performed in PEG solutions of varying concentration, although very often in the dilute or semidilute regime. To prove PEG exclusion near a charged membrane in a more realistic environment, we ran 1 μs all-atom MD simulations in 0.1 M KCl solutions containing 10% PEG600 (w/w). Given the size of the simulation box (∼4 × 10^4^ water molecules), this percentage is roughly equivalent to 120 PEG molecules. Parallel simulations were run by using DPPC and DPPS lipid bilayers. The results are consistent with the simulations of a single PEG molecule. The normalized PEG mass fraction as a function of distance to the DPPS charged membrane (pink symbols in Figure 4C) shows a depleted zone close to the membrane and a nearly uniform concentration region beyond ∼40 Å off the membrane-solution interface. Note that setting 10% PEG over the whole simulation box leads to slightly higher PEG concentrations in the region free of interaction to compensate for the decrease near the membrane. The PEG mass fraction as a function of distance to the neutral DPPC membrane (blue circles in Figure 4C) serves as a reference to separate polymer exclusion caused by steric effects. By converting PEG mass fraction *m*(*z*) into free energy *E* using the relationship *E*(*z*) = −ln[*m*(*z*)/*m*(∞)] and subtracting the DPPC values from the DPPS ones we get a free energy difference Δ*E*(*z*) consistent with the PMF obtained from simulations of a single PEG molecule (Figure 4D). There is a slight difference between the free energy difference obtained in simulations of a single PEG molecule and a PEG solution, possibly due to the crowding effect. This finding is parallel to what is seen in NR experiments, which show less PEG exclusion near the membrane in more concentrated solutions of PEG.

## Discussion

Macromolecular interactions between charged or neutral cosolutes and charged biological surfaces depend both on electrostatic interactions and their coupling to other modes of interaction.^25^ In crowded biological environments, many different interactions are present, and their interplay can result in interesting emergent behaviors. The crowding effect on bimolecular association, ligand-to-surface binding site association, protein folding, and enzyme activity has been the object of experimental and theoretical studies. It is commonly assumed that crowding effects are predominantly entropic in origin, often attributed to a reduction in the configurational entropy of the active macromolecules, i.e., the volume excluded by the presence of crowders,^2^ and work in the direction of protein stabilization. Recently, however, it has been shown that other attractive and repulsive interactions are important^1,26−30^ and in some cases they can be protein-specific.^29^ In this work, we have focused on the interaction of neutral polymers, which are commonly used crowders, with charged biological membranes. This repulsive interaction might have an analogue in the effect of other neutral crowders on charged macromolecules. We have shown that due to sDEP and counterion pressure, the neutral polymer is excluded from the membrane surface to a greater extent than expected from purely entropic effects. Although there are differences between synthetic polymers and biologically relevant crowders,^1^ the underlying interaction mechanisms are quite general, and relevant especially for nanoscale biological macromolecules.

Dielectrophoretic effects have not received much attention for nanoscale particles, for good reason: the electric field gradients required to overcome thermal energy for a nanoscale particle is much higher than the field gradients employed in typical DEP experiments.^31^ DEP is one of the most widely used techniques for the manipulation and sorting of cells and all sorts of bioparticles near the micron scale and larger (colloidal particles, red blood cells, viruses, and large proteins). DEP applications at the nanoscale have evolved alongside the improvements in the design of miniaturized electrodes and microfluidic devices.^32^ For nanoscale experiments, the electric field gradient required to overcome random Brownian motion is ∇*E*^2^ ≈ 4 × 10^21^ V^2^/m^3^.^33^ In state-of-the-art protein DEP,^31^ field gradients are ∼10^24^ V^2^/m^3^ or less. By contrast, the electric field gradient created by a charged DPPS membrane in a 0.1 M salt solution is an astounding ∇*E*^2^ ≈ 10^24^–10^25^ V^2^/m^3^ at a distance between 5 and 15 Å from the charged headgroups.

As for the counterion pressure, an order-of-magnitude calculation suggests that it should also be relevant for nanoscale particles. For example, the total ion excess concentration is ∼0.67 M at a distance one Debye length from the DPPS membrane surface in a 0.1 M KCl solution. Using the van’t Hoff equation for rough calculations, we estimate an osmotic pressure of 1.67 MPa and the corresponding osmotic pressure gradient, i.e., the hydrostatic force per unit volume, as ∼6 pN/nm^3^. For a particle of radius *R*_h_ = 0.7 nm (like PEG600) the total hydrostatic force from counterion pressure is ∼8 pN, while the randomized thermal force would be *k*_B_*T*/(2 *R*_h_) ≈ 3 pN.

The NR measurements provide unambiguous evidence that, indeed, PEG molecules in an ionic solution are excluded from a region next to the lipid polar headgroups to a larger extent in charged membranes than in neutral zwitterionic membranes (Figures 3B–D). Interestingly, positively charged and negatively charged membranes behave alike within the small differences between their respective surface charge densities. Figures 3B–D demonstrate that the depletion layer, although small, is measurable and comparable to the PEG size and the Debye length of the KCl solution. Note that the charged membranes used in the experiments have only 50% of the charge of a pure DOPS or DOTAP membrane, so that the effect would be larger in fully charged membranes (a rough calculation using the analytical expressions predicts an increase of ∼7 Å in the exclusion layer for PEGs with MW in the range 600–2000). The depletion effect becomes much smaller in experiments with a higher PEG concentration. One possible origin for this effect could be that in the semi-dilute regime (Figure S5), molecular overlap matters more, i.e. molecules are no longer interacting with the field independently, but instead have a reduced effective radius. At low salt concentration, the Debye length is significantly longer, the field gradients are reduced accordingly, and the repulsion effect is smaller. The experiments do not *a priori* rule out differences in hydration that account for either the concentration or the salt effect; however, MD simulations do not show any effect of hydration at the distances in question. In particular, the relatively large roughness of the lipid membrane surface is likely to disrupt the collective effects that are responsible for strong hydration layers.^34^ It is also unlikely that phase separation induced by the high salt concentration near the charged surface is responsible for the observed effects, as this effect is not observed at room temperature for NaCl and KCl at sub-molar concentrations.^35,36^

The MD simulations of PEG molecules near zwitterionic, negatively, and positively charged lipid bilayers in 0.1 M KCl solutions convey the same message as the NR experiments. The calculated PMF of a single PEG molecule rises steeply as the polymer is positioned near a charged membrane (using the AWH method), while it increases near a neutral membrane only when confinement of the PEG chain and short-range interactions come into play. Interestingly, the difference in PEG PMF between DPPS (or DPTAP) and DPPC decays with distance to the membrane as predicted by the theory, i.e., Δ*E*(*z*) ∼ exp(−2κ*z*), and it increases with PEG MW, thus yielding a wider depletion zone. Subsequent MD simulations with PEG 10% (w/w) solutions in the same 0.1 M KCl buffer yield parallel differences between PEG mass fraction distributions near neutral and negatively charged membranes. When the mass fraction is converted into free energy to compare single PEG and PEG solution simulations, we see the simulations capture the reduced crowding effect seen in the NR experiments (Figures S2 and S4D).

Recently,^30,37^ several effects of PEGs as crowders in solutions of intrinsically disordered proteins of different net charge have been reported. PEGs induce a reduction in the effective radius of gyration of these proteins compatible with a repulsive interaction. The reduction increases with the protein charge and, most interestingly, also with the PEG MW. These observations are consistent with the increased repulsion of PEGs from charged proteins due to sDEP and counterion pressure but are otherwise difficult to explain.

A recent work on the specific protein stabilizing and destabilizing effect made by sugars as crowders^29^ approaches the enthalpic or entropic origin of the crowding interaction by studying its temperature dependence. According to our analytical expressions, both contributions (from sDEP and counterion pressure) increase almost linearly with temperature at physiological salt concentrations, leading to a prediction that the size of the PEG depleted region should be temperature-independent. However, future work is needed to experimentally support this prediction.

Finally, we can speculate about the possible role of a depletion region near biological membranes in intracellular transport. Due to the crowded conditions and both chemical and morphological complexity of the cell interior, it is not surprising that transport within cells is highly anomalous, characterized even for single species by a wide range of time scales and apparent diffusivities.^38^ Because sDEP and counterion pressure selectively exclude larger particles to larger distances from charged membrane surfaces, such as those found on interior cell membranes, it is tempting to speculate that transport near the membrane surface is enhanced for small- to medium-sized biomolecules and metabolites that are too large to undergo free diffusion in the crowded cell environment but too small to experience significant exclusion effects themselves. *In vivo* observation of such transport could be due to the effects described in this manuscript.

## Conclusions

We present direct evidence for the repulsion of neutral molecules from charged interfaces. The repulsion arises from the extremely high field gradients near a charged interface, which act on neutral molecules directly through surface dielectrophoresis and indirectly via counterion pressure from the electric double layer. Experimental observations of the repulsion of neutral PEG from charged lipid bilayer interfaces using neutron reflectometry are well matched by molecular dynamics simulations. Further, the simulation results scale as expected from analytical expressions for sDEP and counterion pressure. The effects described here have implications for the partitioning of neutral substrates into nanopores and porous materials, drug design and delivery, the activity of proteins near membrane surfaces, and transport of small molecules along the cellular membrane surfaces.

## Experimental Section

### MD Simulation

Poly(ethylene glycol) (PEG) molecule models of various MW (600, 1000, 1540, 2000, 3400 and 6000) were built using the Charmm-gui^39^ service (Polymer builder–poly(ethylene oxide)) by selecting the corresponding number of monomers with the best approximation to the target MW (respectively 13, 22, 35, 45, 77, and 136 monomers for the above MW). Similarly, lipid membrane models were prepared with Charmm-gui of zwitterionic 1,2-dipalmitoyl-phosphatidylcholine (DPPC), negatively charged 1,2-dipalmitoyl-phosphatidylserine (DPPS), and positively charged 1,2-dipalmitoyl-3-trimethylammonium-propane (DPTAP). In all cases the resulting membrane included around 180 lipid molecules distributed over the two bilayers.

VMD^40^ was used to extract and combine the PEG molecules and membranes obtained in the previous steps into a single system. The VMD’s *add solvation box* and *add ion* extensions were then used to add water and K^+^ and Cl^–^ ions to a final ionic concentration of 0.1 M (or as needed) and to achieve an electrically neutral system. The final system box, before minimization and equilibration, had approximate dimensions of 85 × 85 × 265 Å and consisted of a total of approximately 150,000 atoms, including ca. 42,000 water molecules, with minor variations depending on the specific system considered.

The system was then relaxed after an initial minimization and several equilibration steps (two in the NVT ensemble and three additional steps in the NPT ensemble) in which initial position restraints over the membrane and PEG molecules were gradually removed. In all computations, the 2021 version of Gromacs^41^ running on a GPU-CUDA system and the CHARMM36 force field were used.

The final production step (10 ns) was run in the NPT ensemble. A time step of 2 fs was used, with PME electrostatics with a cut-off of 1.2 nm and van der Waals interaction with a Verlet cut-off of 1.2 nm and a force-switch modifier of 1 nm. All H links were constrained with LINCS.^42^ The Nosé–Hoover thermostat was applied during production with a coupling constant of 1 ps and a reference temperature of 298.15 K. A Parrinello–Rahman isotropic barostat with a coupling constant of 5 ps, a reference pressure of 1 bar, and a compressibility of 4.5 × 10^–5^ bar^–1^ was applied. The TIP3P model for water was used for all simulations.

The equilibrated systems as described in the previous procedure were used as input for computing the PMF of a single PEG molecule using the AWH method as implemented in Gromacs.^41^ We selected as a reaction coordinate the distance *z* between the lipid membrane and PEG mass center, with *z* ranging from ∼3.5 to 7 nm, depending on the PEG size. We defined 8 replicas for the AWH with a *per replica* simulation time of 70–200 ns (giving a total simulation of 630–1600 ns per simulated system) and with all the remaining simulation parameters as in the production step. The output was analyzed using the *gmx awh* Gromacs function. The PMF obtained in the presence of a neutral DPPC membrane was subtracted from the corresponding to a charged membrane (DPPS or DPTAP) to eliminate any entropic contribution from the final PMF profile. We assume than the membrane charge is located on the O2L oxygen atoms of the lipid headgroup (CHARMM atom naming convention) both for DPPC and DPPS lipid bilayers, and the CL atom in the case of DPTAP bilayers.

Additional systems were prepared containing 120 PEG molecules and a lipid membrane (DPPC or DPPS) in a simulation box with enough water molecules and ions to simulate a 10% w/w PEG/0.1 M KCl water solution. These systems were run for a sufficiently long simulation time to extract the concentration profile of PEG molecules in the vicinity of the membrane. The PMF was obtained by adjusting the mass fraction profiles (the output of the simulations) to exponential decay from the membrane surface. These simulations allowed us to explore any potential crowding effect into the PMF profile by comparing PMF for a single PEG molecule with that for a 10% PEG solution.

### Neutron Reflectometry

Single-crystal silicon wafers (100, n-doped to a resistivity of 1–100 Ohm cm) of 5 mm thickness and 50.8 mm diameter, polished on a single side, were cleaned in concentrated sulfuric acid and dried with a nitrogen stream. The cleaned, polished surface of the sample wafer was mounted facing a ∼0.16 mm reservoir defined by a 42 mm inner-diameter cylindrical silicone gasket separating the sample wafer from an unpolished backing wafer, also made of single-crystal silicon.^43^ The backing wafer was perforated by single inlets and outlets, which were coupled by IDEX Health and Science (Oak Harbor, WA) flat-bottomed fittings to external tubing for solution exchanges, which were performed using at least 6.0 mL flowing at about 0.5 mL/min. The reservoir volume was estimated to be 0.22 mL.

To prepare vesicles, a solution of lipids in the desired molar ratio was prepared at 5 mg/mL in 2 M NaCl, subjected to at least 40 min of bath sonication, and injected into the sample cell. Incubation proceeded for at least 1.5 h, followed by flushing with pure water to lyse the vesicles via osmotic stress, forming a supported lipid bilayer membrane.

NR experiments were carried out on the LIQREF horizontal reflectometer at the Spallation Neutron Source at the Oak Ridge National Laboratory and on the CANDOR reflectometer at the NIST Center for Neutron Research. In both cases, a polychromatic beam of neutrons impinged on the interface between the polished surface of the sample wafer and the liquid in the sample cell reservoir. The pre-sample collimating slits were chosen to maintain a constant illuminated interface area for each measured angle θ. Post-sample collimation was chosen to allow the entire reflected beam to impinge on the detector, which was positioned at an angle 2θ relative to the incoming beam direction to measure specular reflection. Each reflectivity curve from LIQREF covered a range in scattering wavevector *Q* = 4πλ^–1^ sin(θ) from 0.008 to 0.379 Å^–1^, binned to maintain a constant bin width equal to 2.5% of the bin center.

The reflectivity was calculated as *R*(*Q*) = [*I*(*Q*) – *I*_B_(*Q*)]/*I*_0_(*Q*). Here *I*(*Q*) is the measured count rate (normalized to a much larger monitor count rate to account for fluctuations in beam intensity) under the specular condition. *I*_B_(*Q*) is the background intensity, which arises primarily from incoherent scattering from the liquid reservoir and is calculated by linear interpretation of the intensities measured by the detector at off-specular positions bracketing the specular condition. *I*_0_(*Q*) is the incident beam intensity and is directly measured through the silicon substrate at θ = 0 with the detector positioned in line with the incident beam.

NR data were analyzed using the composition space modeling procedures described previously.^44^ Briefly, the composition space model arranges the known molecular components of the tethered bilayer and protein at the substrate surface; any unfilled space is assumed to be filled with water. Because the nSLD of each component is known or can be estimated from its elemental composition and molecular volume, an average nSLD profile can be calculated as a function of the distance from the substrate surface. This nSLD profile in turn corresponds to a predicted *R*(*Q*) which can be optimized to the experimental data, using as parameters the spatial arrangement of the molecular components. Replacing all H_2_O in the membrane-bathing buffer with D_2_O provides contrast and allows unambiguous determination of the nSLD profile associated with both measured *R*(*Q*) curves by simultaneous optimization of the two contrast conditions.^45^ The volume fraction profile of PEG is described by the expression
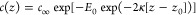
Here, *c*_∞_ is the bulk volume fraction, κ^–1^ is the Debye length, *z*_0_ is the position of the plane of charge, and *E*_0_ is an interaction strength parameter in units of *k*_B_*T* representing the energy of the PEG molecules at *z* = *z*_0_. Where the PEG profile overlaps with other molecular groups, *c*(*z*) is scaled by the available space. The nSLD of PEG was taken to be constant at 0.7 × 10^–6^ Å^–2^. To account for changes in the hydration of the bilayer in the presence of PEG, the separation between the bilayer and the substrate and the distribution of lipids between the inner and outer leaflets of the bilayer were allowed to vary, but the total volume of the bilayer was held constant.

Optimization was performed on a high-performance computing system at the NIST Center for Neutron Research using the DREAM Markov Chain Monte Carlo algorithm^46^ implemented in the software package Refl1D.^47^ Confidence intervals on parameters and model predictions were calculated from parameter distributions derived from 1.4 million DREAM samples after the optimizer had reached steady state.

## Supporting Information Available

Additional details of mathematical expressions, computational methods, and neutron reflectometry results (PDF)
