# Addition to “Charged Biological Membranes Repel Large Neutral Molecules by Surface Dielectrophoresis and Counterion Pressure”

**DOI:** 10.1021/jacs.4c03162

**Published:** 2024-03-22

**Authors:** Marcel Aguilella-Arzo, David P. Hoogerheide, Mathieu Doucet, Hanyu Wang, Vicente M. Aguilella

In page 17 of Supporting Information, we inadvertently overlooked the reference to a paper by Getfert et al., which also uses the integration of the Maxwell stress tensor and the pressure tensor for obtaining an analytical expression of the dielectrophoretic and the hydrostatic force, respectively. It reads:

4. Getfert, S.; Töws, T.; Reimann, P. Reluctance of a Neutral Nanoparticle to Enter a Charged Pore. Phys Rev E Stat Nonlin Soft Matter Phys 2013, 88 (5). https://doi.org/10.1103/PhysRevE.88.052710.

The new ref 4 is quoted in pages 3 and 4 of the Supporting Information.

The authors apologize for this oversight. A corrected Supporting Information is also provided with this Correction. The findings and conclusions of the paper remain unchanged.

## Supporting Information Available

Additional details of mathematical expressions, computational methods and neutron reflectometry results (PDF)

