# Supplementary material for: Charged Biological Membranes Repel Large Neutral Molecules by Surface Dielectrophoresis and Counterion Pressure
Source: J Am Chem Soc. 2024 Jan 16;146(4):2701–10. doi: 10.1021/jacs.3c12348 (PMC10835712; doi:10.1021/jacs.3c12348)
Supplement: Supplementary file 1 — ja3c12348_si_001.pdf [file ja3c12348_si_001.pdf]

Supporting Information for publication

Charged biological membranes repel large neutral molecules by surface dielectrophoresis and counterion pressure.

Marcel Aguilella-Arzo[a,1], David P. Hoogerheide[b,1], Mathieu Doucet[c], Hanyu Wang[d], and Vicente M. Aguilella[a]*.

[a]*Laboratory of Molecular Biophysics, Department of Physics, Universitat Jaume I, 12071, Castellón, Spain*

[b]*Center for Neutron Research, National Institute of Standards and Technology, Gaithersburg, MD 20899, USA*

[c]*Neutron Scattering Division, Oak Ridge National Laboratory, Oak Ridge, TN* 37831*, USA*

[d]*Center for Nanophase Materials Sciences, Oak Ridge National Laboratory, Oak Ridge, TN 37831, USA*.

[1] M.A-A. and D.P.H contributed equally to this work.

* To whom correspondence may be addressed.

Vicente M. Aguilella   Email: aguilell@uji.es

1. Charged membrane induced dielectrophoretic force on a neutral spherical object immersed in an ionic solution (derivation of the expression for F_D).

The classical expression for the DEP force acting on a neutral, homogeneous, spherical particle of radius *a* is [1]:

$$\mathbf{F}_D = 2\pi\,\varepsilon_w \left[\frac{\varepsilon_p - \varepsilon_w}{\varepsilon_p + 2\varepsilon_w} \right] a^3\, \nabla\left(E^2\right) \tag{1}$$

where ε_p and ε_w are the absolute permittivities of the particle and the surrounding medium, respectively. *E* is the modulus of the electric field (or the rms in oscillating fields). The fraction enclosed in brackets is the Clausius-Mossotti factor, related to the effective polarizability of the particle. A first correction to this expression involves solving exactly the electrostatic potential both inside and around the particle. The general solution of Laplace equation for the electric potential within the particle under azimuthal symmetry can be expressed in terms of Legendre polynomials (using spherical coordinates *r* and θ with symmetry around the z axis):

$$\Phi_{\text{int}}\left(r,\theta\right) = \sum_{n=0}^{\infty} \left(A_n r^n + \frac{B_n}{r^{n+1}} \right) P_n\left(\cos\theta\right) \tag{2}$$

The external potential is obtained after solving Poisson-Boltzmann equation in its linear approximation in the region outside the spherical particle. The general solution can be expressed as:

$$\Phi_{ext}\left(r,\theta\right) = \sum_{n=0}^{\infty} \left[A_n I_n\left(\kappa r\right) + B_n K_n\left(\kappa r\right) \right] P_n\left(\cos\theta\right) / \sqrt{\kappa r} \tag{3}$$

where I_n and K_n are modified Bessel functions. For the case of an infinite charged plane with surface charge density σ in a solution of Debye length κ^{-1}, the external potential at distance *z* is given by:

$$\Phi_{ext} = \frac{\sigma}{\kappa \varepsilon_w} e^{-\kappa z} \tag{4}$$

and, after expansion in series, it reads:

$$\Phi_{ext} = \sqrt{\frac{\pi}{2\kappa r}}\, \frac{\sigma}{\kappa \varepsilon_w} e^{-\kappa z} \sum_{n=0}^{\infty} \left(2n+1\right) I_{n+1/2}\left(\kappa r\right) P_n\left(\cos\theta\right) \tag{5}$$

This expression accurately captures the external potential with only a few terms (typically up to n = 3) in the series expansion. Once the electric potential is mapped, we calculate the force on the boundary of the particle by integrating the Maxwell stress tensor, as done in other biomolecular applications as [2,3]

$$\mathbf{F}_D = \int_{S_p} \frac{1}{2}\left(\varepsilon_p - \varepsilon_w\right)\left(\mathbf{E}_o \mathbf{E}_i\right)\mathbf{n}(\mathbf{r})dS \tag{6}$$

where $\mathbf{E}_o$ and $\mathbf{E}_i$ are the electric field outside (o) and inside (i) the particle boundary surface and $\mathbf{n}(\mathbf{r})$ is the unit normal vector pointing away from that boundary. This expression should be further integrated to obtain the DEP force modulus as a series expansion where $\bar{a} \equiv \kappa a$ is the dimensionless particle radius and leads to the following expression for F_D, the z-component of the DEP force, normal to the charged plane:

$$F_D = \sum_{n=0}^{\infty} \frac{4\pi n(n+1) A_n A_{n+1} \varepsilon_w \left(\varepsilon_p - \varepsilon_w\right)\left(\varepsilon_p (n+1) + (n+2)\varepsilon_w\right)}{\bar{a}(2n+1)(2n+3)\left[n\left(\varepsilon_p - \varepsilon_w\right)K_{n+1/2}(\bar{a}) + \bar{a}\varepsilon_w K_{n+3/2}(\bar{a})\right]\left[(n+1)\left(\varepsilon_p - \varepsilon_w\right)K_{n+3/2}(\bar{a}) + \bar{a}\varepsilon_w K_{n+5/2}(\bar{a})\right]} \tag{7}$$

where K_n are modified Bessel functions and the coefficients A_n are functions of σ and κ given by

$$A_n = (n + 1/2)\frac{\sigma\sqrt{2\pi}}{\kappa\varepsilon_p} e^{-\kappa z} \tag{8}$$

The series in Eq. 7 converges rapidly and a few terms (typically up to n = 5) are enough to get the desired accuracy. The dielectrophoretic free energy of the particle as a function of the distance d to the charged interface can be expressed as the work needed to bring the particle from infinity to $z = d$. By grouping into prefactor K_D all terms non-dependent on z we get:

$$E_D = \int_{\infty}^{d} F_D(z)dz = \int_{\infty}^{d} K_D e^{-2\kappa z} dz = -\frac{K_D}{2\kappa} e^{-2\kappa d} \tag{9}$$

where K_D includes the dependence on the other parameters (particle radius, ionic strength, surface charge density and permittivities).

2. Pressure exerted by the Electric Double Layer (derivation of the expression for F_H)

In addition to dielectrophoretic force, the particles in solution are subject to hydrodynamic forces. While the former are generally obtained from the Maxwell tensor (or Maxwell stress tensor), hydrodynamic forces are analogously obtained from the integration of the so-called hydrodynamic stress tensor (or pressure tensor):

$$\mathbf{F}_H = \int_{S_p} \left(-p(\mathbf{r}) + \eta \nabla \mathbf{u}(\mathbf{r}) + \eta \left[\nabla \mathbf{u}(\mathbf{r}) \right]^{\mathrm{T}} \right) \mathbf{n}(\mathbf{r}) dS \tag{10}$$

where η is the viscosity of the fluid, $\mathbf{u}(\mathbf{r})$ is the solvent velocity field and $p(\mathbf{r})$ is the local pressure. For steady-state conditions, mass conservation leads to zero divergence of $\mathbf{u}(\mathbf{r})$ and Eq. 10 simplifies to:

$$\mathbf{F}_H = -\int_{S_p} p(\mathbf{r})\, \mathbf{n}(\mathbf{r}) dS \tag{11}$$

The pressure $p(\mathbf{r})$ follows from solving the Navier-Stokes equation, which in our case simplifies to the form:

$$\nabla p(\mathbf{r}) - \rho(\mathbf{r}) \mathbf{E}(\mathbf{r}) = 0 \tag{12}$$

where $\rho(\mathbf{r})$ is the local charge density due to the mobile ions in solution. By using the Poisson Boltzmann equation for $\rho(\mathbf{r})$ in Eq. 12 the local pressure depends exclusively on the electrostatic field through the relationship [4]:

$$p(\mathbf{r}) = 2 k_B T N_A c_0 \left[\cosh\left(\frac{z e_o \phi(\mathbf{r})}{k_B T} \right) - 1 \right] \approx \frac{-N_A c_0 z_0^2 e_0^2}{k_B T} \phi(\mathbf{r})^2 \tag{13}$$

The expression on the right, valid for low potentials, allows using of the expression for the external potential outside the particle (Eq. 3) and, after integrating over the external particle surface, one obtains:

$$F_H = \kappa^2 \varepsilon_o \varepsilon_w 2\pi a^2 \sum_{n=0}^{\infty} \frac{2(n+1)}{(2n+1)(2n+3)} \phi_n(a) \phi_{n+1}(a) \tag{14}$$

for the z component of the hydrodynamic force, where ϕ_n represents the radial part of the potential outside the particle:

$$\phi_n(r) = \frac{1}{\sqrt{r}} \left[A_n I_{n+\frac{1}{2}}(\kappa r) + B_n K_{n+\frac{1}{2}}(\kappa r) \right] \tag{15}$$

The coefficients A_n are given by Eq. 8 and the coefficients B_n are obtained from the solution of the linear Poisson-Boltzmann equation.

$$B_n = \frac{(\varepsilon_p - \varepsilon_w) n I_{n+\frac{1}{2}}(\kappa a) + \kappa a \varepsilon_w I_{n+\frac{3}{2}}(\kappa a)}{(\varepsilon_p - \varepsilon_w) n K_{n+\frac{1}{2}}(\kappa a) + \kappa a \varepsilon_w K_{n+\frac{3}{2}}(\kappa a)} A_n \tag{16}$$

Alternatively, it is possible to use the expression of the (simpler) potential inside the particle, since both coincide on the surface of the particle (r = a), thanks to the boundary condition on the potential. The corresponding hydrostatic free energy of the particle as a function of the distance d to the charged interface can be expressed as the work needed to bring the particle from infinity to z = d. By grouping into prefactor K_H all terms non-dependent on z we get:

$$E_H = \int_{\infty}^{d} F_H(z)\,dz = \int_{\infty}^{d} K_H e^{-2\kappa z} dz = -\frac{K_H}{2\kappa} e^{-2\kappa d} \tag{17}$$

where K_H includes the dependence on the other parameters (particle radius, ionic strength, surface charge density and permittivities).

3. Relative strength of the two contributions (F_D and F_H).

When using the analytical expressions of the dielectrophoretic and hydrodynamic force acting on PEG molecules in ionic solution near a charged membrane, a question arises whether the dielectrophoretic and the counterion pressure contributions to the overall repulsive force are similar or one of them prevails over the other.

Figure S1 shows the ratio F_H/F_D at the distance to the membrane where total energy becomes 1 k_BT, for ionic strengths ranging from dilute (10 mM) up to concentrated (1 M) solutions and PEG size typical of low-medium MW (roughly 600–6000 Da) near a charged planar surface with σ = 0.34 C/m^2 (this is the typical surface charge density of a negatively charged membrane with

serine polar groups). At physiological concentrations (~100 mM), F_H is several times larger than F_D.

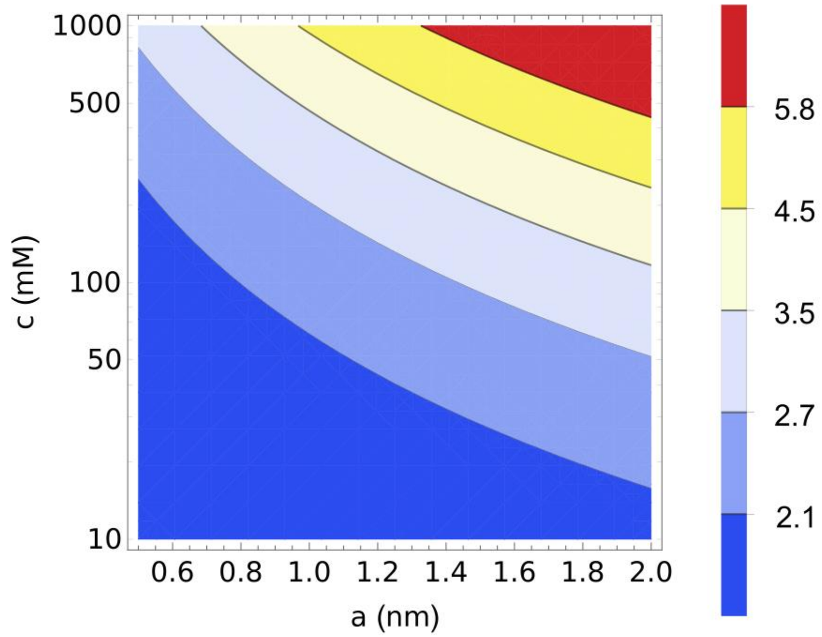

Figure S1. Ratio F_H/F_D between the hydrostatic and dielectrophoretic forces for neutral dielectric particles ($\varepsilon_p = 10\varepsilon_0$) of different size in solutions of varying concentrations near a planar surface with charge density σ = 0.34 C/m^2. The ratio corresponds to the position of the particle where total energy becomes 1 k_BT.

4. MD simulations

System preparation. PEG molecule models of various molecular weights (600, 1000, 1540, 2000, 3400 and 6000) have been prepared using the Charmm-gui [5] service (Polymer builder–Polyethylene oxide) by selecting the corresponding number of monomers with the best approximation to the target molecular weight (respectively 13, 22, 35, 45, 77 and 136 monomers for the above molecular weights). These already solvated PEG models have been downloaded in

GROMACS [6] and/or NAMD [7] format to prepare the systems considered in this work. Similarly, lipid membrane models have been prepared with Charmm-gui with compositions of 100% DPPC, DPPS and DPTAP and 8:2 DPPC-DPPS composition. In all cases the resulting membrane includes around 180 lipid molecules distributed over the two bilayers. These model bilayer membranes have been downloaded from the Charmm-gui service both in GROMACS and/or NAMD native format. VMD [8] was used to extract and combine the PEG molecules and membranes obtained in the previous steps into a single system. The VMD's "add solvation box" and "add ion" extensions were then used to add water and K^+ and Cl^- ions to a final ionic concentration of 0.1 M (or as needed) and to achieve an electrically neutral system. The final system box obtained, before minimizing and equilibration in all cases studied, had approximate dimensions of 85×85×265 Å and consisted of approximately 150,000 atoms, including ~42,000 water molecules, with minor variations depending on the specific system considered. The TopoGromacs [9] was used to convert the combined system CHARMM topology to GROMACS from within VMD. The final systems obtained following the above procedure have been relaxed following a series of steps which are detailed below (Obtained with minor variations of the protocol suggested by the Charmm-gui [5]). In all cases, the 2021 version of GROMACS [6] running on a GPU-CUDA system has been used.

Production. Production was done with not less than 5,000,000 steps with a step size of 2 fs in NPT (10 ns), with PME electrostatics and a cutoff of 1.2 nm and Van Der Waals interaction with a Verlet cut-off of 1.2 nm and a force-switch modifier of 1 nm. All H links have been constrained with LINCS. Nose-Hoover thermostat with coupling constant 1 ps and reference temperature of 298.15 K. Parrinello-Rahman isotropic barostat with coupling constant 5 ps, reference pressure 1 bar and compressibility $4.5 \cdot 10^{-5}$ bar^{-1}.

PMF computation: Accelerated Weight Histogram (AWH). The equilibrated systems as described in the previous procedure were used as input for computing the Potential of Mean Force (PMF) using the AWH method as implemented in GROMACS. We selected as a reaction coordinate the distance *z* between the lipid membrane and PEG mass center, with *z* ranging from ~3.5 to 7 nm, depending on the PEG size. We defined 8 replicas for the AWH with a *per replica* simulation time of ~70 ns (giving a total simulation of ~630 ns per simulated system) and with all the remaining simulation parameters as in the production step. Additional systems were prepared containing 120 PEG molecules and a lipid membrane (DPPC or DPPS) in a simulation box with enough water molecules and ions to simulate a 10% w/w PEG / 0.1 M KCl water solution. These systems were run for sufficiently long simulation time to extract the concentration profile of PEG molecules in the vicinity of the membrane. The PMF was obtained by adjusting the mass fraction

profiles to an exponential decay from the membrane surface. These simulations allowed to explore any potential crowding effect into the PMF profile by comparing PMF for a single PEG molecule with that for a 10% PEG solution.

***Analysis*.** The output was analysed using the *gmx awh* command in GROMACS. The PMF obtained in presence of a neutral DPPC membrane was subtracted from the corresponding to a charged membrane (DPPS or DPTAP) to eliminate any entropic contribution from the final PMF profile. Since the lipid bilayer does not have a well-defined region to ascribe the charge distribution (especially in the case of the charged bilayer: DPPS) it has been considered that it approximately corresponds to the positions of the O2L oxygen of the DPPS/DPPC molecule. The final position considered corresponds to the average position of all O2L oxygens (position averaging) with respect to the center of the bilayer, and along the trajectory considered (time averaging). In the case of DPTAP membranes, the selected atom was the CL from the headgroup. All these analyses have been implemented in a program written in python [10] using the vmd-python module (https://vmd.robinbetz.com/) and several numpy module routines from python that write the results in *.xlsx files (pandas python module) for further analysis and representation.

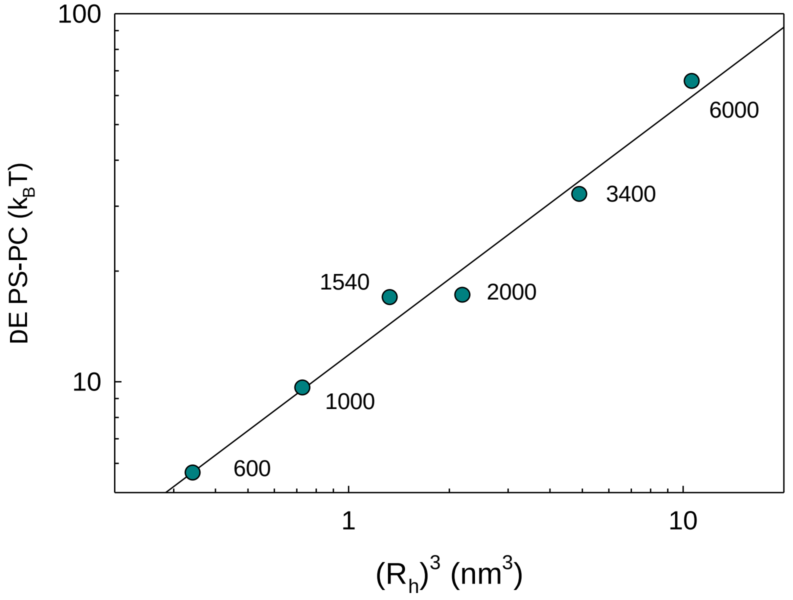

Figure S2. Scaling of the PEG self energy (PMF) obtained from MD simulations with the hydrodynamic radius of the PEG molecule. Values for R_h are taken from [11]. Labels indicate PEG MW.

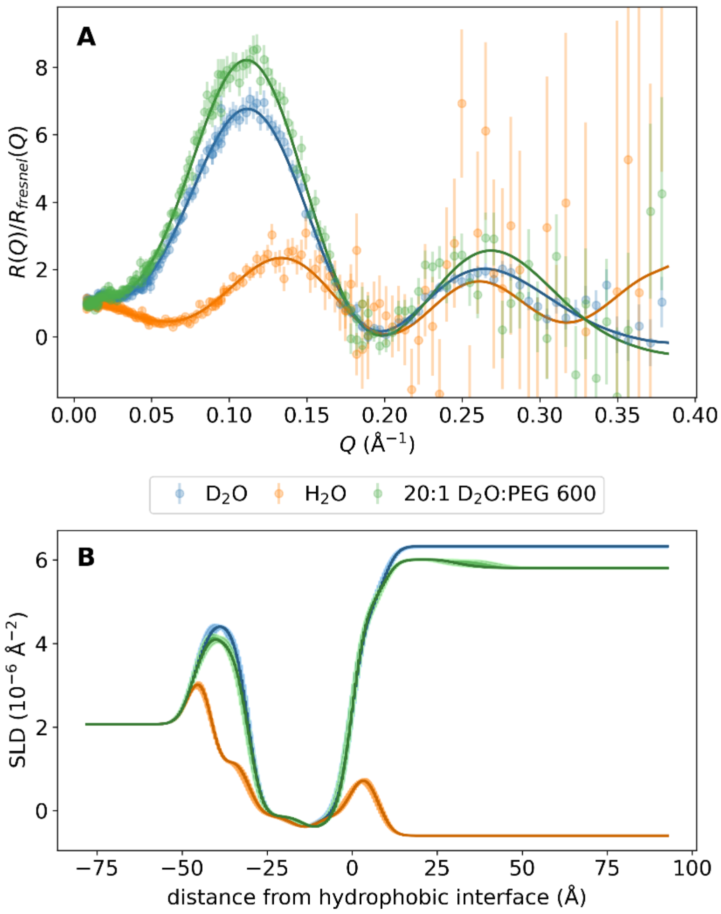

Figure S3. Neutron reflectivity data for a 1:1 DOPC:DOPS lipid bilayer supported on a silicon substrate and exposed to 20:1 D_2O:PEG 600 by weight (100 mM KCl, 10 mM tris, pH 7.4). (A) Neutron reflectivity scaled to the Fresnel reflectivity, i.e. the reflectivity expected between that solution and silicon in the absence of any interfacial structure. Each curve represents the reflectivity of the surface when the reservoir is filled with the solution composition indicated. Error bars are 68% confidence intervals estimated from Poisson counting statistics. (B) Neutron scattering length density (nSLD) profiles of the interface. Shading shows 68% and 95% confidence intervals. Note that exchange of the 20:1 D_2O:PEG 600 solution was not complete, retaining about 5 % H_2O in the flow cell.

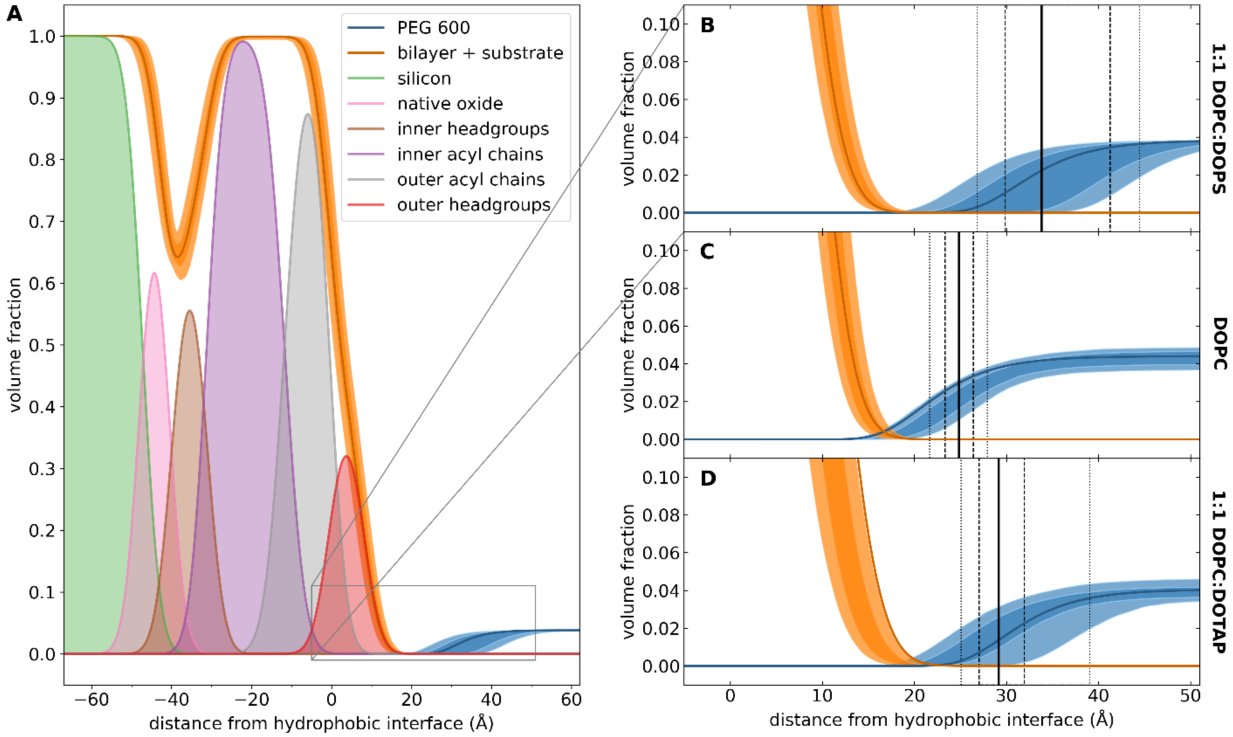

Figure S4. Composition space analysis of NR data for 20:1 D_2O:PEG 600 by weight (100 mM KCl, 10 mM tris, pH 7.4). (A) Volume occupancy profiles of each molecular component. For the bilayer + substrate sum and the PEG 600 profile, shading shows the 68% and 95% confidence intervals, while the dark curve shows the best fit. (B-D) Detail of depletion region near the bilayer surface for three lipid compositions: 1:1 DOPC:DOPS (negatively charged), DOPC (neutral), and 1:1 DOPC:DOTAP (positively charged). Shading represents the 68% and 95% confidence intervals. The vertical solid line represents the median distance from the hydrophobic interface at which the PEG 600 density drops to half its solution density. The dashed and dotted lines represent the 68% and 95% confidence intervals of this value, respectively.

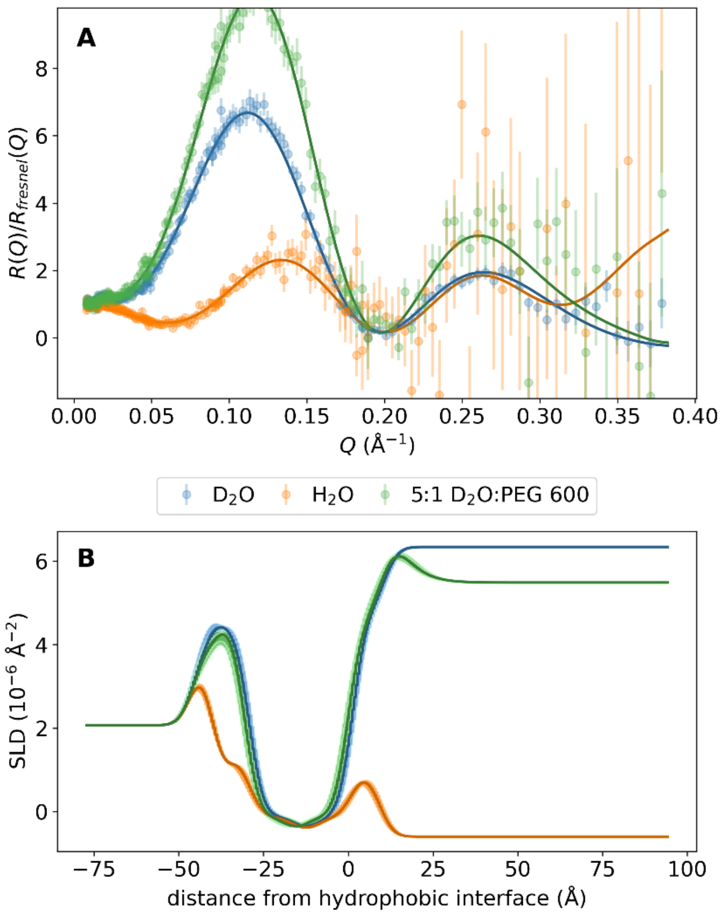

Figure S5. Neutron reflectivity data for a 1:1 DOPC:DOPS lipid bilayer supported on a silicon substrate and exposed to 5:1 D_2O:PEG 600 by weight (100 mM KCl, 10 mM tris, pH 7.4). (A) Neutron reflectivity scaled to the Fresnel reflectivity, i.e. the reflectivity expected between that solution and silicon in the absence of any interfacial structure. Each curve represents the reflectivity of the surface when the reservoir is filled with the solution composition indicated. Error bars are 68% confidence intervals estimated from Poisson counting statistics. (B) Neutron scattering length density (nSLD) profiles of the interface. Shading shows 68% and 95% confidence intervals.

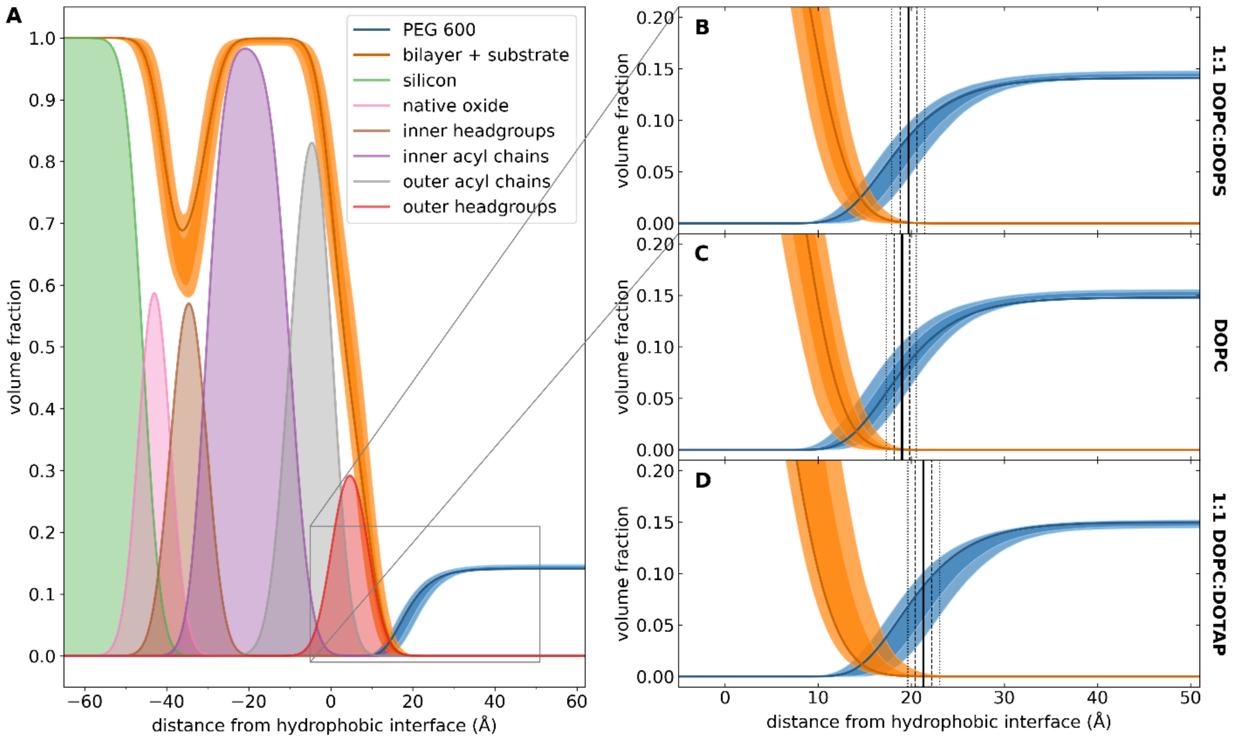

Figure S6. Composition space analysis of NR data for 5:1 D_2O:PEG 600 by weight (100 mM KCl, 10 mM tris, pH 7.4). (A) Volume occupancy profiles of each molecular component. For the bilayer + substrate sum and the PEG 600 profile, shading shows the 68% and 95% confidence intervals, while the dark curve shows the best fit. (B-D) Detail of depletion region near the bilayer surface for three lipid compositions: 1:1 DOPC:DOPS (negatively charged), DOPC (neutral), and 1:1 DOPC:DOTAP (positively charged). Shading represents the 68% and 95% confidence intervals. The vertical solid line represents the median distance from the hydrophobic interface at which the PEG 600 density drops to half its solution density. The dashed and dotted lines represent the 68% and 95% confidence intervals of this value, respectively.

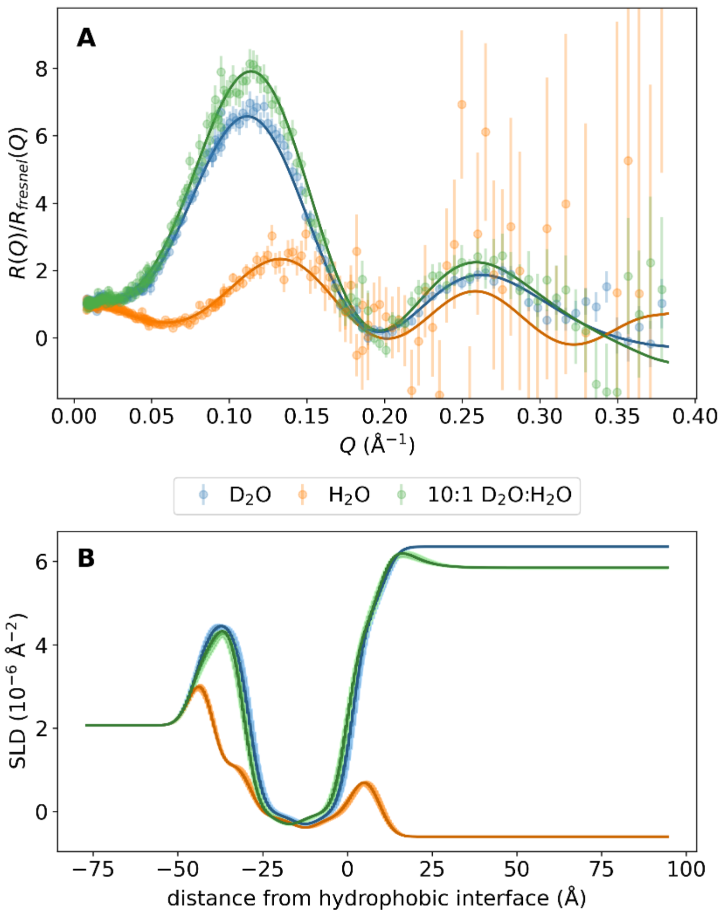

Figure S7. Neutron reflectivity data for a 1:1 DOPC:DOPS lipid bilayer supported on a silicon substrate and exposed to 10:1 D_2O:PEG 600 by weight in low ionic strength conditions (5 mM KCl, 5 mM tris, pH 7.4). (A) Neutron reflectivity scaled to the Fresnel reflectivity, i.e. the reflectivity expected between that solution and silicon in the absence of any interfacial structure. Each curve represents the reflectivity of the surface when the reservoir is filled with the solution composition indicated. Error bars are 68% confidence intervals estimated from Poisson counting statistics. (B) Neutron scattering length density (nSLD) profiles of the interface. Shading shows 68% and 95% confidence intervals.

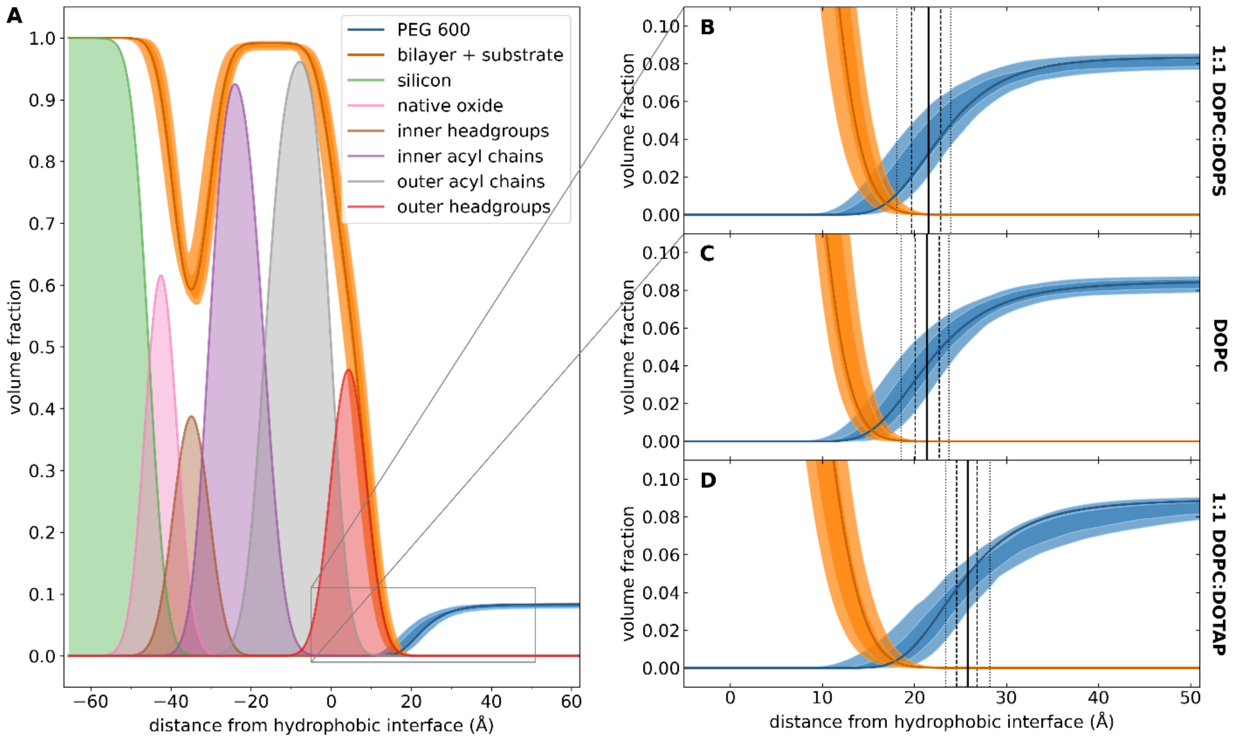

Figure S8. Composition space analysis of NR data for 10:1 D_2O:PEG 600 by weight in low ionic strength buffer (5 mM KCl, 5 mM tris, pH 7.4). (A) Volume occupancy profiles of each molecular component. For the bilayer + substrate sum and the PEG 600 profile, shading shows the 68% and 95% confidence intervals, while the dark curve shows the best fit. (B-D) Detail of depletion region near the bilayer surface for three lipid compositions: 1:1 DOPC:DOPS (negatively charged), DOPC (neutral), and 1:1 DOPC:DOTAP (positively charged). Shading represents the 68% and 95% confidence intervals. The vertical solid line represents the median distance from the hydrophobic interface at which the PEG 600 density drops to half its solution density. The dashed and dotted lines represent the 68% and 95% confidence intervals of this value, respectively.

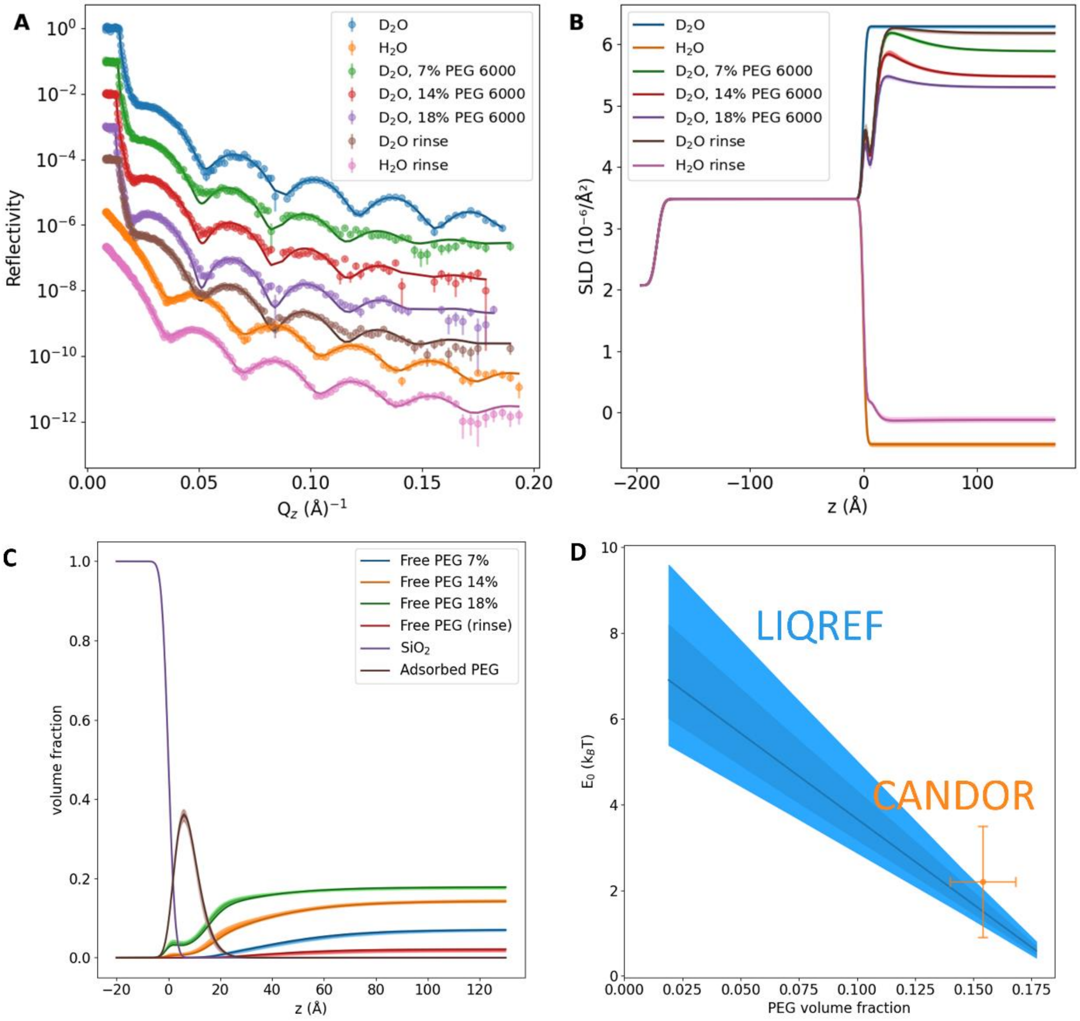

Figure S9. Neutron reflectivity data for a SiO_2 film on a silicon substrate and exposed to PEG 6000 solutions in low ionic strength conditions (5 mM KCl, 5 mM tris, pH 7.4), taken on the LIQREF reflectometer. (A) Neutron reflectivity (unscaled). Each curve represents the reflectivity of the surface when the reservoir is filled with the solution composition indicated. Error bars are 68% confidence intervals estimated from Poisson counting statistics. Solid lines are optimized solutions to the structural model. (B) Neutron scattering length density (nSLD) profiles of the interface. Shading shows 68% and 95% confidence intervals. (C) Volume occupancy profiles of each molecular component. Shading shows the 68% and 95% confidence intervals, while the dark curve shows the best fit. (D) Derived energy of interaction at the SiO_2 surface. Shading shows the 68% and 95% confidence intervals; error bars for CANDOR data on an identical preparation represent 68% confidence intervals.

Table S1. Analytical prediction of the size of the exclusion zone for PEG molecules of varying molecular weight

MW	R_h (Å)	d (Å)
**600**	7	21.2
**1000**	9	25.6
**1540**	11	29.6
**2000**	13	32.5
**3400**	17	38.8
**6000**	22	52.4

SI References

(1) Pethig, R. Review Article—Dielectrophoresis: Status of the Theory, Technology, and Applications. *Biomicrofluidics* **2010**, *4* (2), 022811. https://doi.org/10.1063/1.3456626.

(2) Liu, H.; Bau, H. H. The Dielectrophoresis of Cylindrical and Spherical Particles Submerged in Shells and in Semi-Infinite Media. *Physics of Fluids* **2004**, *16* (5), 1217–1228. https://doi.org/10.1063/1.1649237.

(3) Cai, Q.; Ye, X.; Luo, R. Dielectric Pressure in Continuum Electrostatic Solvation of Biomolecules. *Physical Chemistry Chemical Physics* **2012**, *14* (45), 15917–15925. https://doi.org/10.1039/c2cp43237d.

(4) Masliyah, J. H.; Bhattacharjee, S. *Electrokinetic and Colloid Transport Phenomena*; Wiley, 2006. https://doi.org/10.1002/0471799742.

(5) Lee, J.; Cheng, X.; Swails, J. M.; Yeom, M. S.; Eastman, P. K.; Lemkul, J. A.; Wei, S.; Buckner, J.; Jeong, J. C.; Qi, Y.; Jo, S.; Pande, V. S.; Case, D. A.; Brooks, C. L.; MacKerell, A. D.; Klauda, J. B.; Im, W. CHARMM-GUI Input Generator for NAMD, GROMACS, AMBER, OpenMM, and CHARMM/OpenMM Simulations Using the CHARMM36 Additive Force Field. *J Chem Theory Comput* **2016**, *12* (1), 405–413. https://doi.org/10.1021/acs.jctc.5b00935.

(6) Abraham, M. J.; Murtola, T.; Schulz, R.; Páll, S.; Smith, J. C.; Hess, B.; Lindahl, E. GROMACS: High Performance Molecular Simulations through Multi-Level Parallelism from Laptops to Supercomputers. *SoftwareX* **2015**, *1–2*, 19–25. https://doi.org/10.1016/j.softx.2015.06.001.

(7) Phillips, J. C.; Braun, R.; Wang, W.; Gumbart, J.; Tajkhorshid, E.; Villa, E.; Chipot, C.; Skeel, R. D.; Kalé, L.; Schulten, K. Scalable Molecular Dynamics with NAMD. *J Comput Chem* **2005**, *26* (16), 1781–1802. https://doi.org/10.1002/jcc.20289.

(8) Humphrey, W.; Dalke, A.; Schulten, K. VMD: Visual Molecular Dynamics. *J Mol Graph* **1996**, *14* (1), 33–38. https://doi.org/10.1016/0263-7855(96)00018-5.

(9) Vermaas, J. V; Hardy, D. J.; Stone, J. E.; Tajkhorshid, E.; Kohlmeyer, A. TopoGromacs: Automated Topology Conversion from CHARMM to GROMACS within VMD. *J Chem Inf Model* **2016**, *56* (6), 1112–1116. https://doi.org/10.1021/acs.jcim.6b00103.

(10) van Rossum, G.; Drake, F. L. *The Python Language Reference Manual*; 2009. https://doi.org/10.1159/0000113495.

(11) Kuga, S. Pore Size Distribution Analysis of Gel Substances by Size Exclusion Chromatography. *J Chromatogr* **1981**, *206*, 449–461.
